# Effects of an antenatal mindfulness-based childbirth and parenting programme on the postpartum experiences of mothers: a qualitative interview study

**DOI:** 10.1186/s12884-017-1240-9

**Published:** 2017-02-07

**Authors:** Françoise Roy Malis, Thorsten Meyer, Mechthild M. Gross

**Affiliations:** 1 Midwifery Research and Education Unit, Hannover Medical School, Carl-Neuberg-Str. 1, Hannover, D-30625 Germany; 2 Integrative Rehabilitation Research Unit, Institute for Epidemiology, Social Medicine and Health Systems Research, Hannover Medical School, Carl-Neuberg-Str. 1, Hannover, D-30625 Germany

**Keywords:** Mindfulness, Woman, Antenatal, Childbirth, Postpartum, Perception, Experience, Midwifery, Parenting programme, Shelter

## Abstract

**Background:**

Applications of mindfulness during the perinatal period have recently been explored and appear to offer a decrease in stress, anxiety and depression during this period. However, it still remains unclear what practical use women make of mindfulness during the postpartum period and the mechanisms through which it works. The subjective experience of mindfulness practice by mothers is not fully understood. The aim of the present study was to explore how women enrolled in a “Mindfulness-Based Childbirth and Parenting programme” experienced mindfulness practice during the postpartum period.

**Methods:**

Ten pregnant women over 18 years of age with singleton pregnancies, no diagnoses of mental illness and participation in a “Mindfulness-Based Childbirth and Parenting programme” were recruited to take part in a postpartum interview. Audio recordings of the interviews were transcribed and analysed thematically based on a phenomenological approach. The transcripts of nine interviews were submitted to a coding process consisting of the identification of words, sentences or paragraphs expressing common ideas. These ideas were classified in codes, each code representing a specific description, function or action (e.g. self-perception, personal organization, formal/informal meditation practice). Progressively, a framework of thematic ideas was extracted from the transcripts, allowing the interviews to be systematically organized and their content analysed in depth.

**Results:**

Five themes emerged from the descriptions of practices of mindfulness during the postpartum period: perception of the present moment, breathing, acceptance, self-compassion and the perception of mindfulness as a shelter.

**Conclusion:**

Mindfulness practices during the postpartum period may contribute to a mother’s psychological wellbeing. The perception of mindfulness as a shelter had not previously been reported. Future research could address whether this role is specific to the postpartum period.

## Background

The birth of a child represents a sequence of challenging events, requiring adaptive coping responses to maintain emotional wellbeing [1]. This is of particular interest as stress and anxiety experienced during this period are likely to affect the mother’s mental health [1–3] and impact on the relationship between the mother and her infant [4, 5].

Mindfulness has been applied as a psychological concept in managing stress and anxiety through the practice of meditation [6]. It involves a variety of meditative techniques designed to focus attention on the experience of the present moment in a non-judgemental way. Whether the experience is pleasant or not, the goal is “just to let things be” and “to experience them as they are” [6].

Three major programmes have been developed during the last decades: Mindfulness-Based Stress Reduction (MBSR) [7], Mindfulness-Based Cognitive Therapy (MBCT) [8] and Mindfulness-Based Childbirth and Parenting (MBCP) [9].

While MBSR and MBCT are of interest in human life generally, the MBCP programme has been developed to promote the wellbeing of mothers by reducing the stress related to pregnancy, childbirth and early parenting through mindfulness meditation practice.

Potential applications of mindfulness-based techniques reducing stress and anxiety during the perinatal period have been explored with promising results. Recent studies suggest a relationship between mindfulness and the reduction of perinatal anxiety [10–15], stress [13] and perinatal depression [10, 11, 14, 16, 17].

Mindfulness seems to have an enhancing effect on maternal self-efficacy [12, 13], self-compassion [11, 13], maternal foetal attachment [16] and women’s well-being [10]. It has also been suggested that mindfulness potentially empowers women during birthing, with a higher level of control over the birth process and involvement in the decision-making process during it [18]. It promotes maternal emotional well-being and a quality relationship between the members of the family [14]. In science, great efforts are being undertaken to demonstrate whether the mindfulness-based concept really works [19, 20]. However, there is a lack of research exploring the use and contribution of mindfulness. The mechanisms through which it works during the postpartum period still remain unclear.

The aim of this study is to explore the experience of mindfulness during the postpartum period, with specific attention to its possible effects on the psychological state and wellbeing of the mother.

## Methods

### Setting and planned investigation

The Haute Ecole de Santé in Geneva offers a “*Mindfulness-Based Childbirth and Parenting programme*” [21]. This programme is advertised through the Maternité de Genève (Maternity unit of Geneva), midwives’ and obstetricians’ private practices; it is free of charge. The course is dedicated to pregnant women and their partners. It consists of six three-hour learning sessions, a seven-hour retreat day and one postpartum session. The first part of each session includes mindfulness meditation practice provided by an expert while the second part is an antenatal class offering information on labour and birth, postpartum and breastfeeding by student midwives in their last year programme. During these sessions, different exercises are used to illustrate meditation practices and “moment-to-moment experience*”* promoting awareness of thoughts, feelings, breathing and other physical sensations. The perception of the relationship with the baby is encouraged. No specific technique of breathing is taught, but participants are encouraged to observe their breathing and welcome feelings and sensations that occur. In each session, participants are trained to integrate and accept any discomfort or any unpleasant or painful sensations and to experience them with calm and relaxation rather than with distress and fear. This is to adjust for later labour pain and stressful perinatal situations. Between each class, participants are invited to practice daily mindfulness-based exercises for 30 min while using audio or written support. Mindfulness practice comes in two varieties: formal and informal. Formal practice of mindfulness is the practice of mindfulness meditation, informal practice of mindfulness is the integration of mindfulness behaviour through daily life activities.

### Sampling and recruitment

In order to cover a broad range of post-natal experiences, all pregnant women who signed up for one of the three “Mindfulness-Based Childbirth and Parenting programmes” during the 2012–13 academic year were approached. Women needed to be above 18 years of age and to be free of any severe psychiatric disorder. They needed to attend at least five sessions of the “Mindfulness-Based Childbirth and Parenting programme” and have a delivery planned before the end of May 2013. In view of the international nature of the local community in Geneva and the composition of the international research team we decided to conduct interviews in English; therefore participants were required to have a good command of English. A detailed presentation of the study was given during the fourth session of the programme. The eligible participants gave their oral consent to being included in the study and gave the team permission to contact them during the postpartum period for the interview.

A total of 21 women were initially considered for participation. Of these, 11 were excluded: due to an insufficient level of English (five women), attendance at less than 5 programme sessions (four women), or a due date after May 2013 (two women). The remaining 10 women received an information letter explaining the study with the guarantee of anonymity and confidentiality, a personal data questionnaire and a consent form including their willingness to publish the research results. The women read and signed these documents before the interview started. The research was approved by the Ethics Committee of The University Hospital of Geneva, Switzerland (13–085).

### Characteristics of the participating women

Maternal ages ranged from 25 to 36 years old, with a mean age of 32 (SD +/− 5.0). Of ten women, seven were white Caucasian, two Asians, and one Latin American. Eight mothers were married and all of them were living with their baby’s father. Eight of them held a bachelor’s and/or a master’s degree. Eight mothers were primiparous and two were multiparous. Eight of them had a vaginal birth, two a caesarean section. The ages of the newborns at the time of the interviews ranged from three weeks to four months. Six mothers had histories of mood disorder, including histories of postpartum depression in the cases of the two multiparous women. None of these six women reported any psychiatric disorder in their current pregnancy. Five of the participants had experience of yoga and/or meditation. Five mothers participated in the entire programme, three mothers attended six sessions and two mothers attended five sessions.

### Interview guide and interview performance

A preliminary version of the interview guide was tested with one woman. It was then revised as some questions evoked rather short statements of the interviewee and others were considered as having been too directive. The data of the interviewee was not considered for further analysis. The revised interview guide used open-ended questions to encourage the mother to describe her daily life. It contained questions on experiences of daily life, descriptions of good and/or bad days, and perceptions from the participant concerning the “Mindfulness-Based Childbirth and Parenting programme”, its perceived impact on daily life and its benefits.

Counselling skills like listening, paraphrasing and empathy were used during the interview to promote mutual confidence and a willingness to express experiences and feelings [22].

All interviews took place at the participant’s home during the postpartum period, in the presence of the baby but no other member of the family. The interview lasted 45 to 75 min and was recorded with digital recording equipment (Olympus VN-711PC digital voice recorder). The characteristics of the setting (noise, light, available space) were also noted. The verbatim transcription was effected by the author. Every effort was made to transcribe the recording in its absolute entirety [23], including laughs, cries, hesitations, pauses, attention to the baby, etc. Any changes in the characteristics of the setting were also accurately recorded (additional noises, timing of breastfeeding, time of day, description of the environment).

## Data analysis

### Development of codes and subcodes

This qualitative study was based on a phenomenological analysis of the interviews with the experiences of women in their natural environments [24]. The transcripts were subjected to a coding process, consisting of the identification of words, sentences or paragraphs expressing common ideas [23]. These ideas were classified by being assigned to different codes, each code representing a specific description, function or action (self-perception, personal organization, formal/informal meditation practice, etc.). Within each code, items were named and dated. Progressively, a framework of thematic ideas was extracted from the transcripts, allowing the content of the interviews to be organized systematically and analysed in depth.

All interviews were read carefully four times. During the first reading, an attempt was made to identify each and every description of an experience, and an initial list of codes and subcodes was generated with the support of qualitative data analysis software (MAXQDA 11). During the second and third readings these codes and subcodes were progressively refined, with the addition or suppression of items. This process resulted in a final working list of 11 codes relating to superordinate categories (description of the mother’s daily activities; description of her organization; description of regular activities; description of breastfeeding/bottle feeding; description of difficult moments; resources drawn upon to cope with a difficult moment; mindfulness practice (informal and formal); what mindfulness gives her; perception of her life; perception of the mindfulness-based “Childbirth and Parenting programme”; reasons why she does not apply mindfulness techniques and 195 subcodes. All were colour-coded and systematically defined in the memo section. During the final reading, the entire content of each interview was broken down into the specific corresponding codes and subcodes.

It quickly became apparent that the creation and application of these codes and subcodes was critical for the thoroughness and quality of the analysis. They were based on a close reading of the interviews followed by the recognition of identifiable items of various natures (psychological, perceptional, organizational, behavioural, etc.). The range and number of codes were not restricted, and they were directly extracted from the terms used by the participants themselves. This is in accordance with an inductive approach where the terminology is generated from the participant’s vocabulary, as opposed to a deductive approach where the terminology and codes of the study are pre-defined by the researcher [23].

### Development of central categories

Following the coding phase, each interview was summarized into a case vignette. The purpose of this step was to afford a better perspective as to the interaction of the different codes in the content of a single person’s interview. It converted the transcripts into a more compact, structured and reader-orientated format. All significant elements were preserved and grouped into four categories: initial motivation for the mindfulness programme; perception of the Mindfulness-Based Childbirth and Parenting programme; the woman’s daily routine and organization; formal or informal practice of mindfulness. Systematic comparison of the cases was used to develop the themes that represented the breadth of postpartum experiences of the young mothers.

## Results

Five themes emerged from this analysis of maternal mindfulness experiences.

### Attention to the present moment

A common theme that emerged from the nine interviews was the intention to “pay attention to the present moment”. Five participants described how they enhanced their perception and experience of the present moment towards the newborn baby and their environment. This includes the reporting of enhanced consciousness and the intention to focus on the present moment.

Véréna and Marianne, for example, reported enhanced consciousness towards their newborns and a focus on the present moment:

*A few times per day when I breastfeed … I return myself to the present moment, I look at the trees, I look at my baby, sometimes I close [my] eyes. I try to feel [this instant of] breastfeeding and I re-centre on myself … When I get stressed, or something is bothering me, then it also helps me to calm my spirit and I feel it’s good also for my relationship with my baby. (Véréna)*

*When I’m doing a massage with her, we are really like in a bubble and I really try to feel deeply everything, when I’m doing the massage and yes to really feel everything. (Marianne)*

The idea of paying attention to the present moment was found in situations where the mothers and the newborns appeared to be distracted, especially by media. Attention to the present moment seemed to offer the opportunity for redefining priorities in daily life:

*Margaux is only … four months but she is already very interested in TV and cell phones, it’s terrible. So I really have to turn off everything and just to be with her and to be 100% with her. Bottle is a good time for that, I really try every time to be with her entirely. (Gaëlle)*

The example of Mary shows how she felt that her baby was calling for her attention:

*With the baby, I have realized that I have to be mindful. For example, when she is eating and I am not looking at her and if I am playing with my phone or watching TV, she gets really mad, she is like “I need your attention”, she needs that I look at her, look at her eyes and she eats better. (Mary)*

Paying attention to the present moment is also related to different aspects of the environment, as is shown in these quotes from Gaëlle and Marianne:

*It’s like a medicine … I remember once I was walking on the street in the sun, it was very beautiful, and at this moment I was very conscious … Because I felt very good at this moment and I smiled, two people smiled at me so it is like a transmission …and it is a very good feeling, very positive. (Gaëlle)*

*I really try to feel everything in the moment, the wind, the sun, to hear all the noises, to feel the heat, to look at all the trees around me. (Marianne).*

### Breathing

Five women reported on breathing as a form of resource. Conscious use of breathing appeared to have positive effects on the women, both as a tool in its own right and also integrated into other techniques, e.g. “*the body scan*”, “*the three-minute meditation*”, or focusing on the present moment. Conscious use of breathing enabled the women to keep a clear mind for decision-making, and to accept thoughts, sensations and emotions. It also supported them in the coping with stress and pain.

Marianne described how she experienced and used breathing as a part of both “the three-minute meditation” and “the body scan”.

*When I’m too upset, I take a three–minute meditation just to breathe deeply. I think about my body, my breathing, and then I can start again. (Marianne)*

*First I concentrate on my breathing and I breathe really deeply and then I try to feel all the parts of my body, like a very quick body scan in one minute and then I let all the thoughts, everything that stresses me, all the thoughts I let them come and just I think inside me it’s okay, I have the right to be upset, I have the right to be stressed, I have the right to be tired, I have the right to have enough. (Marianne)*

For Mary too, “*the body scan*” and breathing had become important resources which helped her to direct her thinking as well as to go to sleep.

*I focus on every part of my body. I start with my feet and then go through my legs. I only think about that, so I go through all my body. (Mary)*

*I focus on the part, but I keep my mind on my breathing. (Mary)*

For Laurène, concentration on breathing supported her in taking appropriate decisions.

*It really helps me every day because I have a lot of thoughts in my head and with this exercise I can breathe, and I can say “Okay it is just a thought and this is not reality”, so I concentrate on what is important. (Laurène)*

*This allows me to think and take the right decision … thanks to the breathing, I can think. (Laurène)*

Laurène focused on her breathing when stress was growing. She also concentrated on what she was doing and this seemed to decrease her stress level. She explained:

*Now I am able to stop everything, to stop the train when I feel stressed or anxious. (Laurène) Mindfulness is with me every day, because every day I have some stress moments due to the daily routine … I am using it, when the stress is growing … I breathe … I think that I was unable to concentrate before and I was running everywhere because I did not take the time to focus on myself. (Laurène)*

Denise, when experiencing a stressful situation, tried to shift her attention away from thinking and to concentrate only on breathing and observation. She explained:

*Okay, I don't even say “Don’t panic”, there's no dialogue, it is very quickly a reflex of “Okay, let’s breathe, let’s breathe, let’s be in the present moment.” (Denise)*

*The breathing is just a reminder … Being in the present moment, it’s just observing what’s happening. (Denise)*

For Denise, this reflex of “being there” and living the present moment was a way to protect herself from negative thoughts and feelings such as guilt and worries.

She was just living the present moment, breathing without thinking about past events which might induce guilty feelings, or about the future and its unknown perspectives for herself and her baby. For Denise, breathing was a strategy to shelter herself in the present moment.

Mary described the way she coped with pain which has been caused by a chronic disease. She had become aware of her breathing during the programme and it had become a very precious tool to deal with the pain which she experiences more or less regularly:

*I can really control the pain with the breathing and if I am breathing regularly then [the pain] becomes bearable. (Mary)*

*I imagine a swing inside of me, I can see this swing in my tummy so when I am breathing, the air goes down pass the diaphragm and comes back up. It looks like a hammock. And this allows me to have a very regular breathing, I do not breathe too fast or too slow, but in a very regular manner. (Mary)*

### Acceptance

Five women reported acceptance as a way of behaving and a mechanism by which a stressful element could be neutralized. Mothers accepted their psychological states without judging themselves. They were able to accept that they could not manage and control everything.

Valentine identified feelings of anger, accepted them and let go of them:

*I come inside very easily, it’s like something natural, when I feel a little stressed with children … I feel this anger and I let it go and it’s okay and yes it’s natural. (Valentine)*

She added:

*If I feel stressed and I don’t want to be stressed, I listen to me … First I listen to this emotion, I can see my emotion, just to accept my emotion … I say “I am upset”, and it’s new for me to say that. (Valentine)*

The women seemed to be confronted with societal expectations in terms of functioning well as a mother managing a household. Valentine’s baby was her third and she was aware that she could not manage everything. She explained:

*I let something go and it comes very naturally, you know … Before I was so strict with me, especially with me and I think I probably wanted to be perfect, you know, a perfect woman who can do all together. (Valentine)*

*I’m more in a new reality, more simple you know, I don’t want to be “the sun”, I just want to live my life and don’t think, I am not thinking so much, I’m just living my life. (Valentine)*

Marianne explained that her daughter set a new priority for her. She accepted not being able to do everything:

*I try to see [the entire] situation as if nothing is urgent, even if I’m late, even if I forget something for the baby; it doesn’t matter. I always think: “Okay, when Laurence is well, then everything is okay, the rest is not so important”. And then sometimes I just say “Okay, now I stop for just one minute and I try to do it, to start it again as simple as possible” and often it works. I just say “Okay, I don’t need … to do this or to take this with me, or to wonder what the people will think about me if I’m late” or something like that. It doesn’t matter. (Marianne)*

This went along with feelings that mothers were unable to control everything:

*Now, I’m in this present moment because I accept that I can’t tidy up [my apartment] for the moment, it’s too much. Before, because I wanted to control all of my life, I would be in stress … I accept this reality, I do what I can, in this moment and when it’s possible. I do the essential. (Valentine)*

Laurène too has been more capable of accepting her life as it comes since she introduced mindfulness into her thought process. She reported a rainy morning when she had to walk to the day care centre:

*I said “It is raining, so it is not a problem […], we will take the time.” And this is really not me! If you knew me six months before, I would have said “It is raining, that not my chance” …and then “It is everybody’s fault”. I was not able to put things into perspective and say: “Yes, it is raining and this is life.” (Laurène)*

### Self-compassion/self-kindness

Self-compassion is a psychological process conferring the ability to accept one’s body and mind as they are, acknowledging and tolerating one’s imperfection. Self-kindness confers an attitude of benevolence and positive respect towards oneself. In this study four women described self-compassion and self-kindness as new psychological behaviours in their lives.

Valentine and Marianne reported that they used to be very strict with themselves. Valentine now felt that she expected less of herself. She confided:

*Before, I was so strict with me … I probably wanted to be perfect. (Valentine)*

Marianne reported that she did not judge herself anymore; she was now able to accept herself the way she is:

*I think before I was too, too strong with myself. I always thought everything is my fault. So it is really this non-judgement on myself that I have learned. When something goes wrong, I’m able now to think “Okay it's not my fault” …, it’s okay if I’m not perfect, it’s okay if I’m tired, it’s okay if I’m stressed. (Marianne)*

She also explained that this programme had brought her a lot of peacefulness. Beforehand and during the whole pregnancy she had been wondering if she would be a good mother. She had been afraid of not having enough patience to take care of her baby. She explained:

*My first preoccupation was: “Will I be able to be a good mother?” … When I’m tired, I often have no patience at all, so I was afraid I [would not] be enough patient with my baby … It’s a lot of thoughts like that …and the mindfulness helped me a lot not to judge myself. I think this is the most important thing I’ve learned. (Marianne)*

Self-kindness was also reported as a new feeling. Mothers were more sensitive to the needs of their newborns. Denise developed kindness towards herself and then towards her daughter while participating in the programme. She said:

*It’s thanks to mindfulness that I can be kind to myself now. (Denise)*

*The mindfulness class helped me to be kind to myself so that I can be kind to Jade. (Denise)*

Self-kindness was also mentioned by Gaëlle. Mindfulness allowed her to have an attitude of benevolence towards herself:

*It brings me the possibility … to be sensitive to my needs and to live things more intensively. (Gaëlle)*

Gaëlle explained that mindfulness had allowed her to become a “friend of herself”, giving her a lot of confidence:

*I feel now more confident because during the mindfulness lesson, the meditation taught me more things about myself and I was less and less afraid of myself, because when you know yourself better and better, you begin to become friend of yourself and yes, you feel more confident, I think. That’s the most important thing that mindfulness gave me. (Gaëlle)*

### Mindfulness seen as a shelter

The perception of mindfulness as a shelter was mentioned spontaneously by four mothers. The concept of sustained protection, a trustworthy ally, was a recurrent message in these interviews. Mothers knew that they could rely on mindfulness on a long-term basis.

*Now it is in me, and I know how to do it. … It’s like a shelter, I know that I can do it and take a lot of benefits from that. (Gaëlle)*

*Mindfulness is like a seed: once it has been planted, it’s for life, nobody can take it out of yourself. (Gaëlle)*

Valentine compared mindfulness to a present for her entire life.

*I took this mindfulness course like a present for my life, at this moment … and for the future too… I can use it every day. (Valentine)*

Célia and Marianne both described mindfulness as a resource that they could rely on if they felt the need for it:

*I know that it is here if we need, at some point, to rely on something. (Célia)*

Marianne was very worried about developing a postpartum depression. In fact she was very well during the postpartum period, but she knew that she could always rely on mindfulness should she feel the need for it.

*I know that I can always return to this and …also I think I would try if I feel this depression is coming back to react directly and I think the mindfulness would help me a lot. (Marianne)*

Mindfulness was seen as an available inner resource, a reassuring element for the mothers. They knew they could rely on it if they felt afraid or unwell.

### Difficulties in practising mindfulness

In addition to the five themes, some mothers reported that it was difficult for them to practise either formal mindfulness meditation or informal mindfulness. Two out of nine participants did not practise formal or informal mindfulness in difficult moments.

Célia had an uneventful postpartum period. Although she might have considered the practice of mindfulness at some point, she did not use it:

*I never thought about taking three minutes for me, or just breathing or thinking about my body … I am really in … living things plentifully … with my baby, when I am with him. But in the stressed moments I never thought about breathing, or about my body. (Célia)*

Lauriana did not use mindfulness as a resource even though she experienced some difficult moments:

*I had no possible action to get well, to help the situation. So it is frustrating … I could not be active …I could not find any action to get better. I could not choose, it’s like this. It is also something that we learn, that I learn with my baby that you don’t choose. You just take it, and accept it, and accept the best way you can. But there is nothing you can really do. (Lauriana)*

This situation was perceived by the mother as overwhelming, reaching a threshold beyond which mindfulness could not be practised. This in turn could suggest that a minimal amount of energy is required by the individual to be able to use mindfulness in an organized, structured and efficient way.

Gaëlle, a previously very active mother, reported that since her baby was born it had been difficult to find time for herself:

*Oh yes, yoga, meditation, jogging, yes … Since I gave birth to Margaux, it was not very easy for me to practice mindfulness. (Gaëlle)*

Gaëlle reported feelings of guilt for not being able to practice mindfulness as much as she would have liked:

*That’s the black point of mindfulness, maybe because now I know that I can live things more intensively, but I’m not doing that now, so I feel guilty! (Gaëlle)*

The feeling of guilt is an important element to consider, as it may represent a possible unwanted side-effect of the programme: the induction of negative feelings or self-image if mindfulness is not practiced.

## Discussion

This study adds a new category for the childbirth period: mindfulness experienced postpartum as a shelter. It also confirms already known categories during pregnancy.

The practice of mindfulness during the postpartum period seems to increase mothers’ feelings of wellbeing and to offer strategies to adapt to an uncomfortable situation, such as stress, anxiety or pain. These strategies are described as focusing on the present moment, enhancing consciousness, breathing and behaving with acceptance and self-compassion. These themes have been identified separately with the aim of analysing the thought mechanisms for each of them, but most of the time these themes interact with each other.

### Perception of mindfulness as a shelter

Perception of mindfulness as a shelter was mentioned spontaneously by four mothers. The concept of sustained protection, a trustworthy ally, was a recurrent message in these interviews. Mothers knew that they could rely on mindfulness on a long-term basis. Two of them reported mindfulness as an inner force which could be reactivated on demand. Mindfulness described as a shelter is a previously unreported theme identified in this study. Whether this feeling is specific to the postpartum is unknown and certainly worth further study. Indeed, a feeling of being sheltered thanks to the constant availability of a psychological resource might be particularly useful during the postpartum, a period of high maternal vulnerability where coping resources are essential.

### Perception of the present moment

Perception of the present moment has already been described in the literature as *“*embracing the present” or *“*staying in the present moment” [14, 25]. Duncan describes the perception of the present moment as a core element of informal practice.

In this qualitative study, the awareness of the present moment seemed to allow the mothers to concentrate on themselves, and so to be more receptive to the outer and inner sensation of the instant. This mindfulness state appeared to attenuate daily stress and strengthen the relationship between the mother and her baby. Mindfulness and enhanced perception allowed them to have total presence during feeding and to connect with their babies. This reassuring, connecting dimension appears essential during the postpartum period, with the creation of a secure link of trust with the baby.

### Breathing

The increased perception of the moment is sometimes combined with the use of breathing, but not always. When mothers describe their experience of breathing, it always involves the same sequence of phases: 1) focusing on breathing, 2) focusing on themselves, 3) observing sensations, feelings or emotions, 4) accepting feelings and sensations exactly as they are, and 5) letting them pass. The intention to focus on breathing and the acceptance of feelings and sensations as they are appears to be an effective strategy to adapt to a distressing situation or a painful stimulus. Accepting these feelings is a key element in the regulation of the emotions [26].

### Acceptance, self compassion

In this study, two forms of acceptance have been described. The first form is the acceptance of feelings and emotions as perceived. When participants experienced stressful emotions or upsetting feelings, they observed them and accepted them as perceived. They accepted their psychological state without judgement. Marianne welcomed stressful or upsetting feelings while thinking: “I have the right to be stressed, to be upset, to be tired”. Valentine also accepted these feelings while acknowledging her emotions and accepting them as they came. Acceptance, through a non-judgemental attitude, allowed the women to come to terms with negative emotions such as guilt, self-denial or loss of confidence and self-esteem – all psychological responses associated with anxiety [27, 28]. Self-compassion is an important aspect of mindfulness, as it interrelates with acceptance and perception of the present moment. Once fully perceived and identified, these stressful thoughts against oneself can be positively managed through self-compassion and acceptance, with subsequent improvement of the psychological wellbeing [29]. This opinion is shared by other authors, for which self-compassion without self-judgement appears essential for the maintenance of psychological balance [30].

Self-compassion appears particularly important during the postpartum period, when self-competence and self-esteem are not fully deployed. And yet these feelings are strong contributors to the psychological well-being of the mothers and positively affect their relationships with their babies [31]. In the present study, self-compassion is well described by Valentine and Marianne, who accepted themselves without judgement, and acknowledged and welcomed their imperfections. Both described themselves as having become more tolerant towards themselves and with fewer expectations. The mindful approach also helped them to accept not being able to control the situation in its entirety. A second form of acceptance involves a mechanism of “letting go” by which subjects limit their own expectations towards themselves. Some participants described well this mechanism of “letting go” and their acceptance of not being able to manage or control the entire situation. Valentine reported a lesser degree of self-expectation; she expected less of herself, she gave up trying to completely control her environment.

The transition to motherhood is a challenging process during which the capacity for adaptation and psychological flexibility are potentially reduced, in particular due to rapid and destabilizing changes in the mother’s image, responsibilities and role. Expectations are high and influenced by social and cultural norms [31]. During this period, the capacity to regulate emotions and develop flexibility is essential for the psychological equilibrium of the mother and the relationship with her baby. Kashdan and Rottenberg underline the high prevalence of psychological inflexibility in anxiety disorders [32].

### Mindfulness practice

The results show that mindfulness was used by most of the mothers at various times during the postpartum period. It also became apparent that mindfulness was experienced in different ways and various situations. Indeed, while none of the nine participants described an organized, formal mindfulness meditation as part as their daily routine, the informal practice of mindfulness and its philosophy were well described as strategies to maintain psychological wellbeing. This is of particular interest as we did not find an indication that the informal mindfulness practice experience of mothers with young babies differ from the mothers with older babies. Informal mindfulness practice does not require a specific amount of practice to be efficient.

Difficulties in practising formal mindfulness have been evoked in different studies [12, 13]. This is corroborated by our study, in which the mothers’ tiredness and shortage of time were often reported. One mother reported a feeling of guilt for not practising mindfulness. The feeling of guilt is an important element to consider as it might induce negative feelings as a possible unwanted side-effect of the programme. Young mothers are continuously confronted with images of how to become a perfect mother. Mindfulness ideas might contribute to these images and might magnify already existing underlying feelings of guilt if these mothers feel unable to live up to this ideal.

The practice of formal mindfulness meditation is demanding. Potential difficulties in its daily application should be thoroughly explained in order to avoid any feeling of guilt or regret. It is particularly important to emphasize informal mindfulness through an attitude of openness and acceptance, as it appears to be an important and efficient resource for psychological well-being and deep bonding between the mother and her child. Informal practice requires less time compared to formal meditation. Informal mindfulness should be suggested to mothers if they do not have the time to practice formal mindfulness meditation.

### Limitations of the study

Initially it was planned to include all the women who participated in the Mindfulness-Based Childbirth and Parenting programme from November 2012 to February 2013. Inclusion criteria were attendance at at least five sessions of the programme and the absence of any severe psychiatric disorder. However, as the results needed to be written in English and to avoid translation bias, it was decided to conduct the interviews entirely in English.

If this eliminated any translation bias, it also limited the diversity of the sample, restricting it to French-speaking women with a good level of English. Although the MBCP programme was free of charge, the participants were predominantly from middle to high socioeconomic groups and with a good level of education. The selection of mothers with a good knowledge of English amplified this bias. There was a majority of Caucasian mothers living with their husbands or partners.

The results of this qualitative study need to be interpreted with caution as the psychological experience may have been influenced by the personality and the maturity of the participant more than by the measure itself. Furthermore, the fact that the interviews were not conducted in the participants’ own language might have led to some culturally dependent statements being incorrectly interpreted.

The primary researcher repeatedly had to reflect on her own preconceptions of mindfulness in order to allow for critical or negative experiences and appraisals to emerge during the interviews. The researcher’s preconceptions of mindfulness could have influenced participants’ answers. To avoid this effect, the mothers were asked to narrate their daily experience without relating it explicitly to the mindfulness programme, and the researcher used open-ended questions to reduce the possibility of suggestiveness.

### Future research

It would be of significant interest to seek to confirm the observations made in this qualitative small-sample study and to pursue the exploration of mindfulness by a quantitative study. Such research would have to include a larger sample with greater ethnic and socio-cultural diversity. A multicentre study, with multiple instructors, control groups and blinded researchers would attenuate the above-mentioned bias. It might be interesting to use a control group consisting of pregnant women following a traditional childbirth class programme. A long-term study would also allow an assessment of the participants’ adaptability and coping mechanisms to perinatal stress over time [33, 34].

## Conclusion

We found well-grounded indications that mindfulness is a strong contributor to psychological flexibility mediated via the present moment, breathing, acceptance and self-compassion, and an important source of well-being during the postpartum period. Moreover, mindfulness can be experienced as a reassuring available resource, or shelter, that might lead to an overall decrease in anxiety. This additional psychological resource, which has not previously been described, is worth further study, as it may contribute to the reduction of anxiety and depression in the vulnerable period after giving birth. These results apply specifically to women who had attended a Mindfulness-based Childbirth and Parenting programme.

## Abbreviations

MBCP: Mindfulness-Based childbirth and parenting programme
MBCT: Mindfulness-Based cognitive therapy
MBSR: Mindfulness-Based stress reduction
